# Actigraphy-derived time in bed: it’s time to put this issue to bed

**DOI:** 10.1093/sleepadvances/zpaf045

**Published:** 2025-07-18

**Authors:** Lauren K Hand, Catherine F Siengsukon

**Keywords:** actigraphy, time in bed, sleep

Dear Editor,

Actigraphy has expanded the capacity to objectively measure sleep in natural environments for clinical research. As the understanding of the impact of sleep on human health grows, accurately measuring sleep to account for confounding, mediating, and/or modulating effects becomes increasingly important for research across disciplines. Therefore, consistently defining actigraphy scoring methods for key sleep metrics is vital to maintain generalizability of findings.

Time in bed (TIB) is an important sleep metric as it is an indicator of opportunity to sleep and adherence to stimulus control. TIB is captured in self-report and objective measures and is intended to reflect the amount of time between getting in bed at night and arising in the morning for the last time, regardless of how much time was spent asleep. In actigraphy, TIB defines the “sleep period,” the time in which all sleep metrics are captured, and is used directly to calculate sleep efficiency (i.e. total sleep time/TIB × 100%) [1]. Since actigraphy simply detects movement, the use of sleep logs is recommended for scoring actigraphy to corroborate the algorithmically generated sleep outcomes [2]. However, while TIB initially appears uncomplicated, several variables from the Consensus Sleep Diary [3] may be used to define TIB parameters (e.g. time one got into bed, time one tried to go to sleep, time of final awakening, time one got out of bed) that could result in drastic variability on all subsequently derived sleep metrics. Essentially the discrepancy comes down to whether one should identify the TIB without regard for intention to sleep (i.e. initial time one got into bed to final time one got out of bed) or TIB with intention to sleep (i.e. lights off with intention to sleep to final awakening without intent to return to sleep) for scoring actigraphy. While both may hold merit for investigation depending on the context (e.g. insomnia, bed practices), they are separate constructs that are likely to hold distinct implications for health and disease risk, possibly contributing to inconsistent findings across studies. Recently, the need to reevaluate the use of decades-old algorithms for scoring actigraphy in the age of more sophisticated machine learning has been discussed [4]; however, even a more updated algorithm has shown the accuracy of determining the sleep period is improved with sleep diary corroboration [5,6]. Therefore, it is paramount that investigators clearly define their methods for determining TIB and scoring actigraphy.

Reed and Sacco [7] previously identified a similar issue pertaining to calculating sleep efficiency. The authors suggested that instead of TIB defined as the TIB that might include time spent watching television or reading, TIB (the denominator of sleep efficiency) should only reflect the intention to sleep time that they termed “duration of the sleep episode” [7]. While investigators at times have heeded this advice [8], this is far from the standard almost a decade later. In fact, recent publications of actigraphy data have defined the sleep period (i.e. actigraphy scoring window) using several different sleep diary variables: “bed and rise times” [9] “lights off/lights on” [10], or “time they got in bed to sleep” and “morning wake time” [11]. These methods are all common practice, thus, the issue is not regarding scientific rigor but rather consistency in terminology and ability to compare similarly named outcomes across studies.

The issue of inconsistent parameters for TIB is particularly relevant for research around insomnia as this demographic often participates in non-sleep-related activities in bed, thus producing a larger variation between literal TIB and TIB with the intention to sleep. To illustrate this point, we present baseline actigraphy (ActiGraph Corp, Pensacola, FL, model LEAP) from a participant enrolled in a trial for those with multiple sclerosis and insomnia. The participant was instructed to fill out a sleep log based on the Consensus Sleep Diary that included (1) TIB, (2) TIB with lights out with the intention to sleep, (3) final wake up time in the morning, and (4) time got out of bed in the morning to start the day. Actigraphy was scored using Cole–Kripke [12] and manually entering two different parameters from the sleep log for “In Bed” and “Out of Bed” (terms in ActiLife): TIB without regard for intention to sleep (i.e. TIB to time got out of bed in morning to start the day, Figure 1a) and TIB with intention to sleep time (i.e. time with the lights outs with intention to sleep to final wake up time in the morning, Figure 1b). As demonstrated in Figure 1, scoring using TIB without regard for intention to sleep resulted in higher TIB (876 vs. 596 min), total sleep time (580 vs. 439 min), wake after sleep onset (287 vs. 135 min), and lower sleep efficiency (66% vs. 73%). If the methodological differences are not accounted for, this degree of discrepancy could contribute to confusion in the literature that determine clinical practice, such as the inconsistent findings on the impact of cognitive behavioral therapy for insomnia when using objective sleep measures that are quite robust when measured via self-report [13]. Therefore, standardization in determining TIB parameters for scoring actigraphy are crucial for maintaining data integrity across studies.

**Figure 1:**
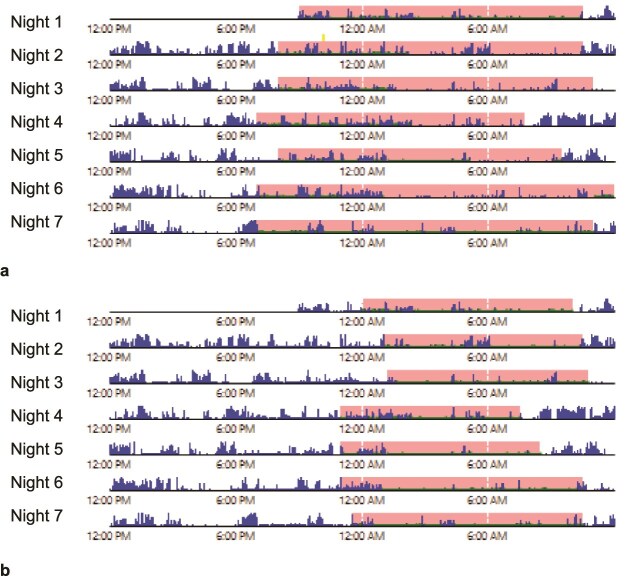
Actigraphy discrepancy based on sleep log extracted time in bed (TIB). The pink regions indicate the sleep period defined by TIB: (a) was scored using literal TIB, defined by the time between first getting into bed and finally getting out of bed per the sleep log. (b) Was scored using TIB with intention to sleep, defined between the time with lights out with the intention to sleep to the final wake up time in the morning per the sleep log. Scoring using literal TIB (a) resulted in higher TIB (876 vs. 596 min), total sleep time (580 vs. 439 min), and wake after sleep onset (287 vs. 135 min), and lower sleep efficiency (66% vs. 73%) compared to scoring using TIB with the intention to sleep (b).

The Society of Behavioral Sleep Medicine issued a guide for actigraphy usage [2]; however, their recommendation on TIB to only include intention to sleep is still not consistently used. For example, the ActiLife 7 User Manual instructs the sleep period to begin with TIB and end with time out of bed [1], seemingly assuming the sleep period defined as such is synonymous with intention to sleep. We recognize that even our definitions of TIB have room for improvement as TIB remains a misnomer as it would still include time getting out of bed in the middle of the night (e.g. stimulus control, urination), and “TIB with intention to sleep” may exclude a portion of time following waking in the morning that may have been spent trying to return to sleep. In fact, the Core Consensus Sleep Diary is similarly limited in capturing the nuance of wake up time [3]. Thus, already established sleep diaries may need amending to explicitly derive intention to sleep, such as using the Expanded Consensus Sleep Diary for Morning [3]. However, particularly in the case of those with insomnia, decisions about which variables to include on a sleep diary must be made with careful consideration not to promote hyper focus on the clock, as it is known to have deleterious effects on sleep [14]. Thus, the sleep research field would benefit from a consensus methodology of scoring actigraphy using sleep diaries with clear parameters for TIB. As an initial step toward reaching a field consensus, clearer methodology needs to be stated in manuscripts or laboratory procedures published for reference. Additionally, to lessen confusion, we propose the term “Time in Bed” be reserved for the period of time from when one got into bed regardless of intention to sleep to when they got out of bed after final awakening and “time in bed with intention to sleep” should be termed the “intention to sleep window” defined as the period of time from when one started intending to sleep to when they stopped intending to sleep. Nuances needed for specific clinical or demographic populations should be clearly described (e.g. shift-workers who may take accidental naps or individuals who sleep in areas that are not their bed at home).

As previously discussed [4] the current age of technology should bring advances in accuracy of accelerometry-derived sleep data. However, the ability to accurately score and replicate sleep data from actigraphy is dependent on a consensus definition and methodology across the field of sleep research for a fundamental sleep parameter: TIB. Most specifically, method transparency and reporting clarity are needed to unify practices and improve data generalizability and comparability, bolstering the rigor of the sleep science field.

## Data Availability

The data used in this editorial is available upon reasonable request to the corresponding author.
